# Antimony resistant bacteria isolated from Budúcnosť adit (Pezinok-Kolársky vrch deposit) in western Slovakia

**DOI:** 10.1016/j.heliyon.2024.e39853

**Published:** 2024-10-31

**Authors:** Hana Majerová, Zuzana Konyariková, Dana Strašiftáková, Christian Puhr, Ivona Kautmanová, Tomáš Faragó, Peter Šottník, Bronislava Lalinská-Voleková

**Affiliations:** a Plant Science and Biodiversity Centre, Institute of Botany, Slovak Academy of Sciences, Dúbravská cesta 9, 845 23 Bratislava, Slovak Republic; b Slovak National Museum - Natural History Museum, Vajanského nábrežie. 2, P.O. Box 13, 81006, Bratislava, Slovak Republic; c Institute of Microbial Genetics, Department of Applied Genetics and Cell Biology, Boku University, Vienna (BOKU), Campus Tulln, Konrad Lorenz Strasse 24, 3430 Tulln, Austria; d Department of Geochemistry, Faculty of Natural Sciences, Comenius University in Bratislava, Ilkovičova 6, 842 15 Bratislava, Slovak Republic; e State Geological Institute of Dionýz Štúr, Mlynská dolina 1, 817 04 Bratislava 11, Slovak Republic

**Keywords:** Bacteria, Iron ochres, Antimony, Arsenic, Resistance, Accumulation, NGS, 16S RNA

## Abstract

Potentially toxic elements (PTE), such as antimony (Sb), are dangerous putative contaminants for ground and surface waters around abandoned mines and ore deposits in Slovakia. Nearby mines antimony is commonly coprecipitated in ochre sediments precipitated from Fe-rich drainage waters and, therefore, these sites function as natural scavengers of this metalloid. Bacteria are well known to contribute to the process of redox state maintenance, biosorption and bioaccumulation of antimony and, consequently, to antimony precipitation or release from iron oxides complexes. Here we isolated 48 bacterial strains from circumneutral hydrous ferric oxides (HFO) rich iron ochres accumulated in the waters running from tailing pounds nearby Budúcnosť mine, Pezinok, Slovakia and polluted with high, but fluctuating, concentrations of antimony (130 μg/l Sb in water and 2317 mg/kg Sb in iron ochre in average). The isolated strains were V1-V9 16S rRNA sequenced and the resulting taxonomic affiliations of isolated strains were compared with taxonomy assignments obtained by V4 16S rRNA next generation sequencing approach, including two independent NGS analysis pipelines and different taxonomy classifiers ((IDTAXA (RDP, GTDB, SILVA, CONTAX), MEGAN (NCBI), RDP a SILVAngs). A Sb resistant subgroup of isolated strains (*Pseudomonas* A60B, *Pseudomonas* A59, *Pseudomonas* A28, *Aeromonas* A21, *Aeromonas* A13, *Aeromonas* A60A, *Acinetobacter* A14, *Buttiauxella* A58, *Shewanella* A20A a *Yersinia* A68), well growing at high Sb concentration (300 mg/l Sb), was tested for an ability of the strains to retain Sb from cultivation media. Based on ICP-MS measurements of the dried biomasses we concluded that all the strains can retain antimony from growth media to some extent, with strains *Shewanella* A20A, *Buttiauxella* A58, *Yersinia* A68 and *Aeromonas* A60A being the most effective.

## Highlights

• Antimony contaminated iron ochres are rich in metal(loid)s-metabolizing bacteria.
• Bacteria isolated from Sb contaminated iron ochres are tolerant to high doses of Sb.
• Seven bacterial strains can retain antimony from media in their biomass.
• *Shewanella*, *Buttiauxella* and *Aeromonas* are the most effective in Sb accumulation.

## Graphical abstract

**Figure:**
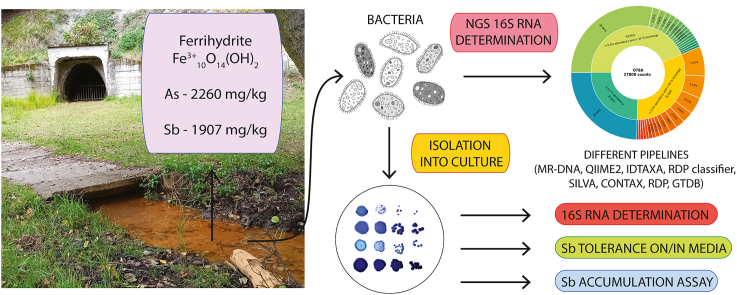

## 1 Introduction

Antimony is a potentially toxic element occurring naturally in many foods, but it may cause significant damage to human health at higher concentrations [1]. In contaminated areas, it enters the food chain either directly, via its solubility in water, or through plants, fungi and bacteria and their subsequent consumption by animals (invertebrates and vertebrates). Therefore, antimony must be considered a risk factor that seriously endangers the functionality and health of ecosystems in a long-term view.

Substantial deposits of antimony (Sb) and its smaller occurrences are located in various zones of the Western Carpathians of Slovakia [2]. Antimony is mined mostly from hydrothermal ores where the mineral stibnite (Sb_2_S_3_) usually dominates. Other primary Sb sulfides, such as berthierite (FeSb_2_S_4_), gudmundite (FeSbS), or sulfosalts may be present in smaller amounts.

Stibnite is commonly co-occurring in association with other sulfides (e. g. arsenopyrite (FeAsS), pyrite (FeS_2_)) and, due to the aerobic oxidation, they are weathering to a limited number of secondary minerals. The oxidation of sulfides usually leads to a release of metal(loid)s, including antimony, into aqueous media, and causes many problems such as water, soil and stream sediments contamination.

Common natural Sb scavengers are HFOs, also called hydrous ferric oxides and iron (III) oxide-hydroxides. Hydrous ferric oxides (HFO) are a family of X-ray amorphous substances of which physical (e.g., surface area) and chemical (e.g., water content) properties vary widely. The lack of a well-defined periodic structure makes the characterization and comparison of HFO samples difficult, although structural models are being developed. Natural HFO is usually referred to as ferrihydrite, a ferric oxyhydroxide mineraloid with small particle size (in the range of 2–20 nm), variable and uncertain chemical composition, physicochemical properties, and structure. Because of the usually small particle size of HFO, the adsorption capacity is high and adsorption may significantly impact the thermodynamic properties of such materials [3]. Sorptive properties of HFOs depend on the pH values of the surrounding water, the chemical composition of the water, and the ratio of the amount of dissolved trace metals to the amount of hydrous iron oxides [4,5]. HFOs harbor a remarkable ability to sequester Sb from the environment by adsorbing antimonate (Sb^V^) and antimonite (Sb^III^) ions on its surface (in some cases even into the structure) under the neutral and low acidic conditions [6,7], which is the case of most groundwater and soil water conditions. Importantly, the redox state of metals in HFO complexes, and Sb adsorption to HFO minerals, are not stable, but they are constantly changing, either as the result of diverse geochemical interactions or as result of interactions with diverse living organisms, mainly bacteria, algae, and fungi [8].

Microorganisms inhabiting iron ochres are well adapted to this extreme environment and many of them, as we have concluded previously, are metabolically active over metal(loid)s accumulation, sorption and redox state maintenance [[9], [10]]. It is very important to understand microbial community structure variation in response to the presence of Sb and associated elements. Many bacteria were reported and some also isolated from Sb polluted water and soil environments [8,[11], [12], [13], [14], [15], [16], [17], [18]] and references therein [[19], [20], [21], [22]], and references therein). Several regular geochemical factors were suggested to play key role in microbial community structure variation in Sb polluted environments, including pH, Eh, SO_4_^2−^/S^−^ and Sb gradients [[23], [24], [25], [26], [27], [28], [29]]. Possible geochemical and biochemical microbial Sb interactions were recently reviewed [8,18] and include Sb detoxification, reduction, oxidation, sequestration, mineralization and methylation. Under physiological circumneutral conditions the antimony is present as uncharged Sb(OH)_3_ in solution and, due to its structural similarities with glycerol, it can be transported into the cells by glycerol transporters [30]. Intracellular Sb^III^ is subsequently exported from the cell by trivalent metalloid/H^+^ antiporters to protect and detoxify the cell [31,32]. The genes responsible for Sb^III^ resistance are induced by increased concentrations of Sb^III^, were reported to be located on both chromosomes and plasmids [17,33,34] and can be easily horizontally transported throughout the bacterial population by means of plasmid transmission, but also by phages [22]. Alternatively, the Sb^III^ inside the cell can be converted to volatile methylstibnite species and released from the cells [8,35]. The aerobic route of Sb^V^ detoxification is largely unknown, but, if it is similar to As^V^ detoxification, Sb^V^ would be imported by phosphate transport systems and converted to Sb^III^ by intracellular reductases [8] and then treated as already outlined. Another route of bacterial Sb reduction seems to be widespread under anaerobic conditions, where the Sb acts as an electron acceptor in dissimilatory respiratory pathway [15,20,[36], [37], [38]]. The Sb reduction can be further enhanced by sulfur reducing bacteria (SRBs), which convert sulfate to sulfide in a process coupled to Sb^V^ reduction [20,39,40]. Even the Sb reduction coupled to sulfide oxidation by *Desulfurivibrio* spp. was reported recently [41]. Numerous studies demonstrate that indigenous microorganisms associated with Sb mine soils are capable of Sb^III^ oxidation, and potentially contribute to the speciation and mobility of Sb *in situ* [[17], [18], [19],^21^,[42], [43], [44], [45]], and references therein). Sb-oxidizing bacteria use Sb^III^ as an electron donor and oxygen under aerobic or nitrogen (or other alternative) under anaerobic conditions as electron acceptors, resulting in the Sb^III^ oxidation to Sb^V^ by periplasmic aerobic As^III^ oxidase, but alternative cytoplasmic Sb^V^ oxidase and monooxygenase also exist [18,[46], [47], [48]], and references therein), however, the fate of Sb^V^ species generated by these enzymes might be diverse.

Besides redox reactions coupled to Sb transport in and out of the cell, and influencing mainly the Sb mobility, bacteria are also supposed to be capable of biosorption and bioaccumulation. The mechanisms involved could either be extracellular accumulation/precipitation, cell surface sorption/precipitation or intracellular accumulation [49]. Although there are missing or very sparse reports of antimony cell surface sorption and intracellular accumulation, they can be expected to occur based on observations with other metal(loid)s. They can be sequestered by chelating substances like extracellular iron-chelating rock solubilizing siderophores or intracellular metal-inducible metallothioneins [[50], [51]]. In case of cell surface sorption, the metal(loid) interacts with the negatively charged functional groups present in biomolecules of microbial cell wall surfaces, such as hydroxyl groups, phosphate groups, carbonyl groups, etc. [[49], [52]]. Bacteria can also increase the sequestration of metal(loid) ions by the means of bioaccumulation on or within their cell walls, both in particulate and insoluble forms. The most essential constituents in this process are exopolysaccharides (EPSs) [52], constitutively produced by certain bacterial cells (e.g. *Pseudomonas*, *Lactobacillus*, *Streptococcus* etc.). Exopolysaccharides are mainly composed of complex high molecular weight organic macromolecules like polysaccharides along with smaller proportions of proteins and uronic acids [52]. Although primarily a defense mechanism of bacterial cells, exopolysaccharide production is associated with biofilm production and is essential for biomineralization of metal ions, including arsenic [[37], [49]]. Only sparse reports for exopolysaccharides and cell walls bound antimony are known up to date and these were reported for *Acinetobacter* sp. JH7 [53] and *Desulfovibrio vulgaris* Hildenborough [54].

Some bacteria were reported to contribute to Sb precipitation and biomineralization. Wang et al. [39] for the first time demonstrated the feasibility of using sulfate reducing bacteria (SRB) to convert sulfate ions in Sb mine drainage into sulfides and cooccurring metal-sulfide precipitation by SRBs is considered to be a promising method for removing Sb from wastewater [40]. Here, formation of sulfides results in Sb^V^ to Sb^III^ reduction and subsequent precipitation of stibnite at pH 7.0 and pH 9.0. Sb-reducing bacteria, such as *Shewanella* sp. CNZ-1 [55], *Desulfuribacillus stibiiarsenatis* MLFW-2 [56,57] and *Sinorhizobium* sp. JUK-1 [36], were later shown to produce biologically generated secondary minerals, e. i. Sb_2_O_3_ or Sb_2_S, during the reduction of Sb^V^ to Sb^III^. Or, similarly, antimony acclimated and sulfate rich wastewater cultures precipitating Sb_2_O_3_ or Sb_2_S had increased population of *Geobacter* and *Pseudomonas* species compared to the wastewater control, suggesting that these bacteria might be responsible for Sb precipitation [58]. Jia et al. [59] reported the ability of *Shewanella oneidensis* strain MR-1 to adsorb and reduce antimony and convert it to SbFeO minerals in the presence of Fe^III^. Similarly, *Sinorhizobium* sp. GW3 was reported to couple Sb^III^/Fe^III^ oxidation to denitrification and Sb bearing Fe-biomineral production [60]. Overall, these studies suggest the feasibility of using bacteria in removing elevated Sb from mine waters, either by redox reactions chemically transforming harmful contaminants into innocuous or less toxic compounds that are more stable, less mobile or inert, or by its sorption, precipitation or biomineralization [[49], [61]].

In the context of iron ochres microbiome characterization, the purpose of this work was to study the extremophilic microbial community of ochreous precipitates at the efflux of Budúcnosť mine near Pezinok in western Slovakia. This site possesses well developed, sun exposed, ochreous sediments polluted with elevated concentrations of antimony and arsenic and represents a putative site for future bioremediation experiments. The aims of this investigation were, therefore, to assess archaeal and bacterial taxonomic affiliations and their relative abundances at this site by the means of next generation sequencing, to isolate Sb resistant bacterial strains, to maintain stable bacterial cultures and characterize them in respect to their ability to tolerate and retain Sb in their biomass, and to compare the bacterial taxonomic affiliations of NGS with bacterial isolation approach.

## 2 Materials and methods

### 2.1 Description of the study site

The Pezinok-Kolársky vrch deposit is located in the Malé Karpaty Mountains, the westernmost mountain range of the Western Carpathians (Slovakia). The ore deposit is hosted by Variscan granodiorites and regionally metamorphosed rocks, that consists of metapelites with zones of black shales and metabasic rocks [62]. The zones contain commonly stratiform pyrite-pyrrhotite mineralization and later hydrothermal ores [2]. The earlier stages of mineralization produced gold-bearing pyrite and arsenopyrite, and subsequently Sb ores, which comprise stibnite, but also relatively abundant berthierite, gudmundite, native antimony and primary kermesite and valentinite. The Sb mineralization occurs as quartz-carbonate (mostly Fe-dolomite) lenses, veinlets, nests, and impregnations in the host rocks. The ore grade is variable, and the chemical compositions of the ores are in the range of 1.2–2.5 wt% Sb, 0.8–1.3 wt% As, and 1.8–2.0 wt% S [63].

The hydrothermal Sb mineralization was mined at the Pezinok deposit between 1790 and 1992, with its most intensive period in 1940. A flotation processing plant has operated since 1906. The ore was ground to a particle size of 40–90 μm and Sb sulfides were partially extracted in the flotation process. A significant fraction of the Sb sulfides and most of the Sb oxides were lost to the waste, which was deposited in two large tailing impoundments [64]. These impoundments together with several adits outflows are the main sources of arsenic and antimony contamination in the area.

### 2.2 Sample collection

Samples of iron ochre and corresponding water were collected from the mine drainage water in front of the Budúcnosť adit (Fig. 1). The exact locality was selected thoughtfully after long term observation. The reasons for the selection were: huge amounts of precipitates present the whole year, elevated concentration of antimony and arsenic in Fe ochre and coexisting water, and direct influence on the surrounding ecosystem.

**Fig. 1:**
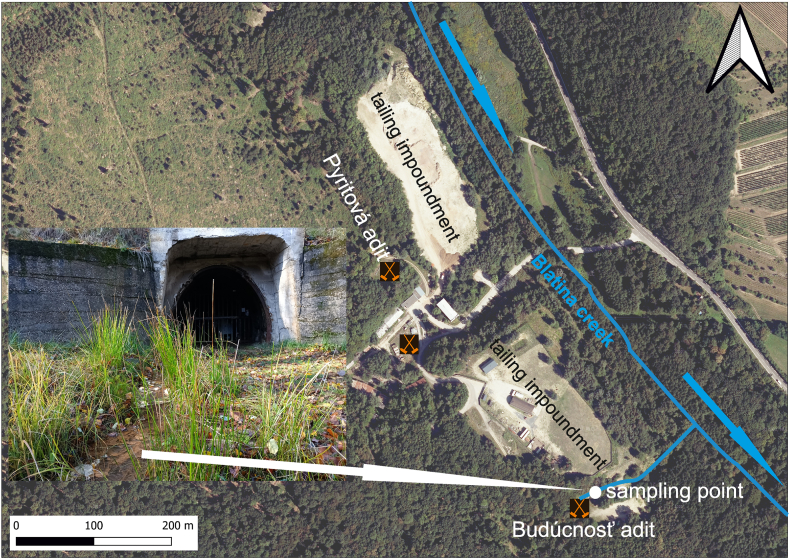
Orthophoto map of the abandoned Sb deposit Pezinok-Kolársky vrch with a sampling point marked.

Collected water samples were filtered through 0.45 μm filter, stored in sterile sampling bottles and transported to the laboratories at 4 °C. One aliquot of the water samples was acidified with ultrapure nitric acid for the determination of major and trace metal(loid)s. Samples of ochre precipitates for geochemical and mineralogical analyses were collected using plastic scoops and put into PTFE bottles. The ochre precipitates were first sieved through 0.16 mm sieve to rid the samples of any residual organic matter (foliage, roots, etc.) and stream sediments. Afterwards, the precipitates were dried in the laboratory dryer at the constant temperature of 40 °C to a stable weight.

Samples of ochre precipitates for microbial study were collected using sterile scoops and gloves, placed into sterile tubes, transported in mobile freezer at −10 °C to laboratory and stored at −45 °C until further processing.

The map of the sampling site was created with the freely available program Quantum GIS 3.16 [65]. It was visualized on georeferenced raster base topographic maps provided by the Geodetic and Cartographic Institute in Bratislava, freely available on the ZBGIS map portal [66]. The background map layer was imported into the QGIS program in the S-JTSK/Krovak East-North coordinate system (EPSG:5514).

### 2.3 Water chemistry

Temperature and EC, pH and Eh were measured in the field at the time of sampling using a WTW Multi 350i instrument equipped with TetraConÒ325 electrode, SenTixÒ41 electrode, and a WTW pH 340i instrument with SenTixÒORP electrode, respectively.

Chemical analysis of water samples was conducted by EL spol s.r.o. accredited laboratories. Samples were collected and processed in accordance with the requirements of the laboratory.

Inorganic elements in water samples were measured mostly by ICP-AES (modelsVarian Liberty 200 and Varian Vista AX) or by AAS (model Varian SpectrAA 220). Arsenic and Sb concentrations were determined by HG-AAS (Varian SpectrAA 220 and hydride generator VGA76). Sulfates and chlorides were determined by ion chromatography (IC Dionex-120). The concentration of NO_2_, NO_3_ and PO_4_ was determined by UV/VIS spectrometry. Instrumental accuracy and precision were monitored using blind duplicates (n = 6) and found to be within the range of ±10 % or better. Concentration of HCO_3_ was calculated as total alkalinity multiplied by molar mass of HCO_3_.

### 2.4 Geochemical and mineralogical analysis of ochre precipitates

The concentrations of selected elements were measured using the ARL Quant’X (Thermo Scientific Inc, USA) EDXRF spectrometer at the laboratory of the Slovak National Museum in Bratislava, Slovakia. Prior to the analysis 5 g of grinned samples were mixed with 1 g of cellulose and pressed into pellets (pressure of 160 kN). Excitation rays are produced by an air-cooled X-ray tube (Rh anode, 40 W maximum power, 4–50 kV anode voltage, 0.02–1.98 mA anode current) and the emitted X-rays are detected by means of a Peltier cooled Si (Li) detector (15 mm 2 crystal area, 3.5 mm crystal depth and 155 eV energy resolution at the 5.9 keV Mn Ka line), and a pulse processor. Certified reference materials (NSC DC73043, CRM016-50, BCR-176R and BCR-320R) were used for proper instrument calibration. All the measurements were performed in vacuum.

X-ray powder diffraction analysis (XRD) was performed at the Earth Science Institute of the Slovak Academy of Sciences using the Philips PW 1710 X-ray Diffractometer, employing CuKα radiation, graphite monochromator, and a scintillation detector. The results were interpreted using the X'pert highscore plus 2.0.1 software.

### 2.5 DNA extraction, preparation of 16S rRNA gene V4 amplicons for illumina Miseq sequencing and bioinformatic analysis

Approximately 2 ml of thawed and well vortexed sample was taken for further processing. First, the sample was centrifuged at 14 000 rpm for 5 min at the room temperature to remove excess water. Then it was dried at 37 °C for 20 min in a Concentrator plus/Vacufuge® plus (Eppendorf). DNA was extracted from the dry sample with DNeasy PowerSoil Kit from Qiagen according to the manufacturer's protocol and the final DNA quality and concentration was checked using 1 % agarose gel electrophoresis and spectroscopy. The bacterial 16S rRNA gene region V4 was amplified using the modified primers illcus515 (5′-GTGYCAGCMGCCGCGGTAA-3′) and new806RB (5′-GGACTACNVGGGTWTCTAAT-3′) [67,68]. Sequencing and library preparation was performed at MR DNA (www.mrdnalab.com, Shallowater, TX, USA) on a MiSeq following the manufacturer's guidelines and internal lab protocols. For taxonomic purposes the sequences were processed in several different pipelines.

1. Sequence data were processed using a proprietary analysis pipeline (MR DNA, Shallowater, TX, USA) described in Leach et al. [69] and it is referred to as MR DNA pipeline in this text. In summary, sequences were joined, sequences shorter than 150 bp and sequences with ambiguous base calls were removed. Sequences were quality filtered using a maximum expected error threshold of 1.0 and dereplicated. Resulting sequences were denoised and chimeras were removed to produce zOTUs. Final zOTUs were taxonomically classified using BLASTn against a curated database derived from NCBI (www.ncbi.nlm.nih.gov) and compiled into designated taxonomic levels (Suppl. C). The most relevant are identity and OTU tables. The identity tables show taxonomic levels based on the percent homology to reference sequence; the taxa with 97 % identity and higher were resolved to species level, with 95 %–97 % identity to genus level, with 90 %–95 % identity to family level, with 85 %–90 % identity to order level, with 80 %–85 % identity to class level and with 77 %–80 % identity to phylum level based on corresponding OTU tables. Tables named ‘species’, ‘genus’, ‘family’, ‘class’, ‘order’ and ‘phylum’ show the most related BLAST results to queried OTUs, but homology of the sequences is diverse. In OTU tables percent homology, e-values and bitscores of individual zOTUs can be found. In table All_OTUs all zOTUs generated in the experiment are shown, including those of eukaryotes and archaea, that were excluded from the other tables.

2. Sequences were processed using QIIME2-2022.8 [70] based pipeline. The raw sequence data were first demultiplexed using the q2-demux plugin followed by quality filtering and denoising by DADA2 plugin [71]. In summary, sequences were joined, trimmed of primers and truncated, so that all sequences shorter than 220 (forward) and 200 (reverse) were removed. Sequences were quality filtered using maximum expected error threshold of 2.0, dereplicated, denoised using default method ‘independently’ and chimeras were removed using default method ‘consensus’. The resulting sequences were summarized by q2-feature-table plugin and used as input in the following taxonomy classification procedures:

A. Classification with IDTAXA classifier [72] against all available 16S RNA databases, i.e. GTDB 16S (revision 207) -unmodified, RDP 16S (version 18) -unmodified, SILVA SSU (138) -modified and CONTAX 16S (version 1) – unmodified, with default confidence threshold of 60 %.

B. Classification with RDP Naive Bayesian rRNA Classifier Version 2.11 (training set version 18) [73] with default confidence threshold of 80 %.

C. Classification with SILVAngs pipeline [74].

D. Classification using BLASTn against 16S ribosomal RNA sequences (Bacteria and Archaea) at NCBI (https://blast.ncbi.nlm.nih.gov/) followed by analysis of BLASTn generated xml file with MEGAN V6.24.16 [75].

### 2.6 Bacterial cultivation

For bacterial cultivation either plates with TSA medium (1.5 % peptone from casein [VWR Chemicals], 0.5 % peptone from soy [VWR Chemicals], 0.5 % NaCl [Slavus], 1.5 % agar) or liquid TS medium (1.5 % peptone from casein, 0.5 % peptone from soy, 0.5 % NaCl), were used. The media were prepared either with standard distilled water or when appropriate the sterile filtered water collected at the sampling site was added instead. For a long time storage an overnight inoculum cultivated at 25 °C, in TS media was mixed with sterile 50 % glycerol (1:1) and stored at −80 °C. When appropriate, KSb(OH)_6_ [SigmaAldrich] was added to the media before autoclave sterilization to achieve desired concentration of Sb. To prepare the liquid media supplemented with Sb, first, the stock solution containing 4 g/l of Sb in TS media was prepared and autoclaved, then, after 1 day at room temperature, the undesired crystals were filtered out and the resulting medium was used in the experiments as the medium with the highest concentration of Sb. The other liquid media for growth assays and Sb removal assays were prepared from this filtered stock by dilutions. The actual Sb concentrations were determined by ICP-MS in each experiment and their average values in 2x diluted stock were 299.7 mg/l ± SD 10.5 mg/l and therefore 300 mg/l value is used as reference in graphical representations and the text.

### 2.7 Isolation of bacteria from river water and sediment

The samples for isolation of bacterial strains were transported and stored at 4 °C. Before the isolation the samples which consisted of river water and ochre-sediment were vortexed and several dilutions were made. 100 μl of each dilution were plated on TSA. Part of the media was made with river water and sludge to see if traces of antimony might influence the bacterial composition. Plates were incubated at 20 °C, 25 °C and 30 °C for 3 days. Each day single cultures appearing were selected and passed on new TSA plates. The selection was performed due to differences in morphological features.

### 2.8 Molecular typing, phylogenomic and taxonomical characterization of isolated strains

For barcode sequencing of isolated strains, a fresh single colony from overnight TSA plate was spread-inoculated onto TSA plate and incubated overnight at 25 °C. The resulting culture was used for colony PCR with general purpose 16S ribosomal RNA gene primers: **27F** (AGAGTTTGATCMTGGCTCAG)/**805R** (GACTACHVGGGTATCTAATCC) and **515F** (GTGCCAGCMGCCGCGGTAA)/**1492R** (CGGTTACCTTGTTACGACTT). The PCR conditions were as follows: Initial denaturation for 5 min at 95 °C followed by 30 cycles of denaturation for 30 s at 95 °C, annellation for 30 s at 50 °C (for 27F/805R) or 30 s at 49 °C (for 515F/1492R), elongation for 45 s (for 27F/805R) or 1 min (for 515F/1492R) at 68 °C, ended by final elongation for 10 min at 68 °C. Reactions were run using the Hot Start *Taq* 2X Master Mix [NEB]. The same primers were used for sequencing. The resulting sequences were combined into one sequence encompassing variable domains V1 to V9 of the 16S rRNA, aligned and clipped at the 5′ and 3’ ends, where needed. The redundant sequences were grouped together and only one representative sequence for each group was used in the final alignment (Suppl. D). A representative sequence from each group was BLASTn classified against 16S ribosomal RNA sequences (Bacteria and Archaea) at NCBI (https://blast.ncbi.nlm.nih.gov/) and the most representative database sequence for each group was added to alignment. Finally, the gapless sequences were aligned and distances were calculated by Clustal Omega (clustalo version 1.2.4, EMBL-EBI, [76]) and the resulting Neighbour joining tree was visualized by iTOL [77]. In parallel the gapless sequences used in final alignment were classified by IDTAXA classifier against RDP 16S (version 18) -unmodified 16S RNA database to obtain comparable classification with NGS data.

### 2.9 Estimation of Sb tolerance on solid media and bacterial morphology

The initial estimation of Sb tolerance was done by drop method on TSA plates. Fresh overnight inoculum cultivated at 25 °C in TS media from stock cultures was used for the drop experiments. OD_600_ of the overnight cultures was estimated and the samples were diluted to OD_600_ = 1. Then 5 μl of the samples adjusted to OD_600_ = 1 and 10 000x and 100 000x dilutions were dropped on TSA plates containing 0, 0.1, 0.5 and 1 g/l of Sb (supplemented as KSb(OH)_6_). The plates were then incubated at 25 °C and bacterial growth was estimated after 24 h. Then the plates were incubated for another 24h and the control containing 0 g/l of Sb was used to determine basic culture morphology: color, surface and texture.

### 2.10 Estimation of Sb tolerance in liquid media

The experiments for estimation of Sb tolerance in liquid media were started by diluting overnight cultures to OD_600_ = 0.1, followed by cultivation at 25 °C with shaking at 230 rpm. The growth of cultures was monitored by measuring OD_600_ in 1-h intervals.

### 2.11 Estimation of Sb retention from culture media by isolates

The experiments were started by inoculating fresh overnight bacterial suspension to 200 ml of liquid TS medium containing either 0 g/l or 300 mg/l of Sb to OD_600_ = 0,15. The cultures were then incubated at 25 °C with shaking at 150 rpm for 16 h. An aliquot of each medium was separated and frozen prior to the experiments and they were used as controls. In parallel with the experiment, an aliquot of TS medium with 300 mg/l of Sb, incubated and treated in the same manner as the bacterial cultures, was used as a control in each experiment. This was to assay for possible spontaneous Sb precipitation during the time course of the experiment. At the end of each experiment the samples were collected by centrifugation at 8000×*g*, 4 °C for 5 min. The supernatants were collected, and the pellets were washed 2 times with 4 ml of TS media and dried. The resulting supernatants were pooled and filtered through a 0.22-μm filter (Frisenette) and stored for analysis. The collected samples, i.e. cell pellets, supernatants and controls, were all analyzed by ICP-MS (EL spol. Ltd.) to determine the concentration of Sb. The amount Sb retained with biomass was expressed either as Sb mass per 1 kg of dried biomass or it was recalculated to the actual mass of dead cells obtained.

To determine the viability of individual strains at the end of each experiment, i.e. after 16 h of incubation with Sb, a small aliquot of each culture was diluted and plated onto TSA plates in triplicates. The number of CFU for each aliquot was determined for each strain, and their viability in media with 2 g/l was expressed as a percentage of CFU counts of the same strain cultivated without Sb.

### 2.12 Accession numbers

All sequencing reads are available from the NCBI SRA database (bioproject number PRJNA854326) under accession number SAMN29427984. The strains Aeromonas sp. A21, Aeromonas sp. A13, Aeromonas sp. A60A, Acinetobacter sp. A14, Buttiauxella sp. A58, Shewanella sp. A20A, Yersinia sp. A68 are available in PCM-POLISH COLLECTION OF MICROORGANISMS, Ludwik Hirszfeld Institute of Immunology and Experimental Therapy, Polish Academy of Sciences, Rudolfa Weigla 12, 53–114 Wrocław under PCM-accesion numbers B/00510, B/00511, B/00512, B/00513, B/00514, B/00515, B/00516.

## 3 Results

### *3.1* Geochemistry and mineralogy

As we found out during previous complex monitoring of the locality, surface and ground waters in the area of the studied deposit were of the Ca-Mg-HCO3-SO4, Ca-Mg-SO4-HCO3, and Ca-Mg-SO4 types [78]. Water samples were characterized by circumneutral pH values despite the process of sulfides decomposition in the environment of mine adits and tailings impoundments, as the acidity generated by the decomposing sulfides is neutralized by abundant carbonates (calcite and dolomite) within the ores.

Chemical compositions of studied HFO sample and coexisting water are listed in Table 1. Analyses from 2002 to 2023 are also mentioned in order to determine whether significant differences in chemical composition occur at the sampling site in time. Water which coexisted with studied HFO was rich in sulfates (335 mg/l). Increased concentration of Ca (120 mg/l) correlated well with high amounts of present carbonates (and their dissolution). Iron content was significantly high and the concentrations of PTE such as As and Sb were elevated and we did not observe a big difference in their concentration over time. Arsenic concentration varied from 50 μg/l (2002) to 130 μg/l (2023) and Sb concentration varied from 88 μg/l (sample 078, 2021) to 130 μg/l (2002).

**Table 1:** Chemical composition of HFO's and coexisting water samples (sample O78 used for this study is in bold).

Sample	pH	conductivity	Fe	Al	As	Sb	Ca	SO_4_^2−^/S_HFO
		μS/cm	mg/L	μg/L	μg/L	μg/L	mg/L	mg/L
**2002_water**	7.3	∗	∗	∗	50.00	130.00	∗	307.00
**078_2021_water**	**7.8**	**1231**	**6.29**	**23.70**	**57.00**	**88.00**	**120.00**	**335.10**
**2023_water**	7.6	1534	**8.66**	110.00	134.00	107.00	70.10	186.10
			**mg/kg**	**mg/kg**	**mg/kg**	**mg/kg**	**mg/kg**	**mg/kg**
**2002_HFO**	∗	∗	74987.00	∗	19785.81	2895.48	995.30	∗
**078_2021_HFO**	∗	∗	**53369.00**	**∗**	**2260.00**	**1907.00**	**15829.00**	**394.00**
**O80_2022_HFO**	∗	∗	**57389.00**	**∗**	**3376.00**	**2149.00**	**13819.00**	**635.00**

Arsenic concentration in HFO changed significantly over time, the sample from 2002 contained 19785 mg/kg of As and the recent sample 3376 mg/kg. Antimony concentration in HFO is more stable and varied from 1907 mg/kg (sample 078, 2021) to 2895 mg/kg (2002).

According to powder XRD analysis, iron ochre samples were made of nanoscale iron oxyhydroxide ferrihydrite in its most simple 2-line form.

### 3.2 Metagenomic analysis

We employed a metagenomic approach targeting the V4 16S RNA genomic region of environmental DNA to identify ∼250 bp long barcode sequences in our sample O78A. We analyzed 28 961 demultiplexed and denoised sequences (counts) generated by MR RNA pipeline and identified 529 independent zOTUs. Bacteria accounted for 96 % (i.e. 27 808 counts) of analyzed sequences. Only 109 counts (0.38 %) were assigned to archaea and were excluded from further analysis. This analysis, but also our previous results [10], indicate that archaea either do not tolerate the conditions well or that they are not detected properly by our 16S RNA analysis.

**Fig. 2:**
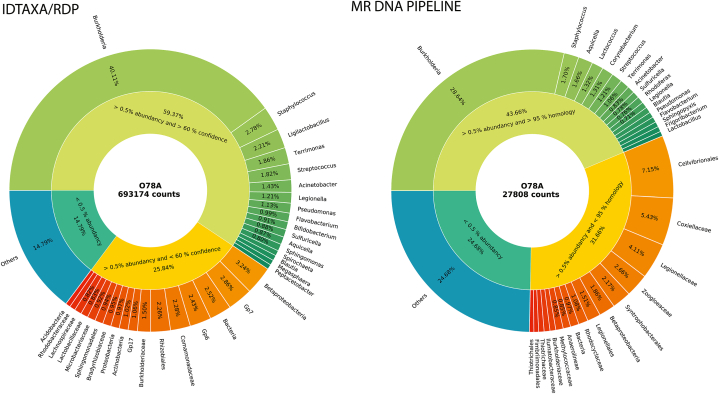
Plots comparing the taxonomy generated by different classification approaches. See text for details. Greens show the most abundant genera detected (more than 60 % confidence at genera level in IDTAXA/RDP or more than 95 % sequence homology in MR DNA pipeline) with relative abundance above 0.5 %, reds show the most abundant taxa other than genera with relative abundance above 0.5 %. Blues show all taxonomy units with relative abundance below 0.5 % summed together.

Using the QIIME2 based pipeline we analyzed 693174 demultiplexed and denoised bacterial sequences grouped into 829 independent ASVs (alternative to zOTUs). The Qimme2 generated ASVs were used as inputs in different 16S RNA taxonomy classifiers, as outlined in section Methods. The classification outputs for individual ASVs and different classification pipelines were compared (Suppl. A.). The comparison of these approaches for 20 most abundant ASVs is available in Table 2. As can be seen from these tables the classification ranks differ substantially between the individual pipelines (e.g. ASV 1 in Table 2), nevertheless, they share some common trends, i.e. the poor classification of certain ASVs (e.g. ASV 2, 3, 6, 7, 8, 15 in Table 2) and more detailed and comparable classification of some other ASVs (e.g. ASV 5, 9, 11, 12, 13 in Table 2). To compare the overall performance of individual classification pipelines, the ASVs with the same taxonomy outputs were summed together, grouped based on their overall abundancies and taxonomy ranks, and plotted as can be seen in Fig. 2 and Suppl. B. The highest portion of abundant sequences (i.e. sequences with overall abundancy higher than 0.5 %) assigned to genus taxonomy level were obtained with IDTAXA/CONTAX (60.91 %) and IDTAXA/RDP (59.37 %) classifiers. The lowest portion was obtained with IDTAXA/GTDB (10.24 %) classifier and NCBI/MEGAN classification pipeline (10.55 %). Intermediate results were obtained with IDTAXA/SILVA (21.10 %) and with the RDP (38.26 %) classifiers.

**Table 2:** Comparison of taxonomies assigned to 20 most abundant ASVs generated with Qiime2 pipeline by respective taxonomy classifiers (see text for details). The full list of ASVs can be found in Suppl. A.

ASV	IDTAXA/RDP taxonomy	IDTAXA/GTDB taxonomy	IDTAXA/SILVA taxonomy	IDTAXA/CONTAX taxonomy	NCBI/MEGAN taxonomy	RDP classifier taxonomy	SILVA taxonomy	counts
1	**Burkholderia**	Bacteria	Gammaproteobacteria	Burkholderia	Burkholderiaceae	Burkholderia	Bacteria	160923
2			Bacteria		Gammaproteobacteria	Bacteria	Bacteria	41906
3			Proteobacteria		Gammaproteobacteria	Gammaproteobacteria	Bacteria	15130
4	**Gp7**	UBA5066	Subgroup 7		Bacteria	Acidobacteria	Subgroup 7	11858
5	**Staphylococcus**	Bacteria	Staphylococcus	Staphylococcus	Staphylococcaceae	Staphylococcus	Bacteria	11232
6		UBA5158			Gammaproteobacteria	Bacteria	Bacteria	9433
7		Bacteria	Ellin6067	Bacteria	Betaproteobacteria	Betaproteobacteria	Ellin6067	6851
8			Proteobacteria		Gammaproteobacteria	Gammaproteobacteria	Diplorickettsiaceae	5974
9	**Terrimonas**	JJ008	Terrimonas	Terrimonas	Terrimonas	Terrimonas	Bacteria	5605
10			Bacteria	Bacteria	Aquicella siphonis	Gammaproteobacteria	Aquicella	5463
11	**Ligilactobacillus**	Bacteria	Lactobacillus	Lactobacillus	Lactobacillaceae	Ligilactobacillus	Bacteria	4427
12	**Actinobacteria**	Corynebacterium	Corynebacterium	Corynebacterium	Corynebacterium kroppenstedtii	Mycobacteriales	Corynebacterium	4236
13	**Acinetobacter**	Neisseria	Acinetobacter	Acinetobacter	Proteobacteria	Acinetobacter	Acinetobacter	4031
14	**Pseudomonas**	Gammaproteobacteria	Pseudomonadaceae	Pseudomonadales	Pseudomonas	Pseudomonas	Bacteria	3935
15		Ilumatobacteraceae	CL500-29 marine gr.		Ilumatobacteraceae	Bacteria	CL500-29 marine gr.	3930
16	**Sphingomonadales**	Proteobacteria	Sphingomonadaceae	Sphingomonadales	Sphingomonadales	Sphingomonadales	Sphingomonas	3896
17	**Microbacteriaceae**	Microbacteriaceae	Microbacteriaceae	Microbacteriaceae	Microbacteriaceae	Microbacteriaceae	Glaciihabitans	3744
18	**Betaproteobacteria**	Burkholderiales	TRA3-20	Betaproteobacteria	Betaproteobacteria	Betaproteobacteria	Bacteria	3703
19	**Sulfuricella**	Sulfuricella	Sulfuricella	Sulfuricella	Betaproteobacteria	Nitrosomonadales	Sulfuricella	3666
20	**Burkholderiaceae**	Comamonadaceae	Comamonadaceae	Comamonadaceae	Burkholderiales	Comamonadaceae	Bacteria	3656

**Fig. 3:**
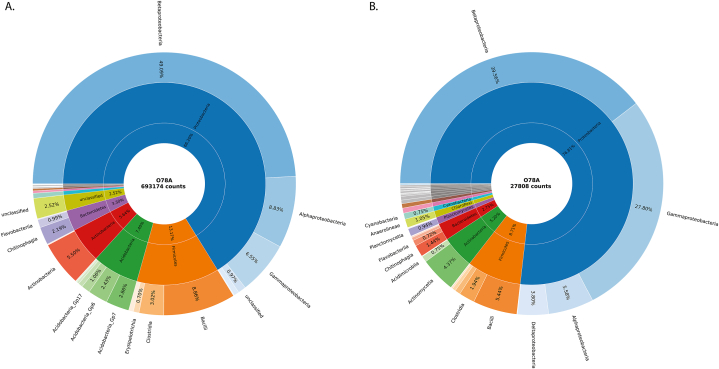
Plots showing relative abundance of bacterial phyla and classes in the sample O78A with IDTAXA/RDP taxonomy (A) and MR DNA taxonomy (B).

The results obtained with MR DNA pipeline, as well as NCBI/MEGAN classification, are based on NCBI data, BLASTn algorithm and percent homology scores and are principally different from those outlined in the previous sections (Fig. 2, Suppl. B. and Fig. 3). The MR DNA pipeline taxonomy was also built from different representative sequences (i. e. MR DNA zOTUs), and therefore the results obtained via this pipeline are not directly comparable with those that we generated with other taxonomy pipelines. But here, using the logic outlined in section Methods, we can state that 43.66 % sequences were assigned to the genus taxonomy level having their partial abundances higher than 0.5 % and percent homologies higher than 95 %.

Although the core-genome alignment derived GTDB database is considered to have the best up to the date taxonomy, the low portion of taxonomy labelled ASVs makes it an unfavorable option in our hands. The RDP 16S RNA database is derived of an authoritative reference taxonomy based on Bergy's Manual of Systematic Bacteriology and this, together with the good performance of IDTAXA/RDP classifier on our data set, makes it the most promising classification option.

For the reasons outlined above, taxonomies assigned to our data by IDTAXA/RDP classifier and by the MR DNA pipeline will be discussed in detail (data are presented as IDTAXA-RDP classifier results/MR DNA pipeline results respectively). Proteobacteria accounted for 66.20 %/76.8 % of detected bacterial sequences and according to both taxonomies create the major bacterial phylum at this locality (Fig. 3). They included four classes: Betaproteobacteria (49.09 %/39.6 %), Gammaproteobacteria (6.55 %/27.8 %), Alphaproteobacteria (8.83 %/5.6 %) and Deltaproteobacteria (0.64 %/3.8 %). The second most abundant bacterial phylum was Firmicutes (13.17 %/8.7 %), mainly represented by classes Bacilli (8.86 %/5.4 %), and Clostridia (3.02 %/1.9 %), followed by Actinobacteria (5.64 %/5.2 %), mainly represented by Actinobacteria/Actinomycetia (5.5 %/4.4 %), and by Bacteroidetes (3.3 %/2.7 %), mainly represented by Chitinophagia (2.19 %/1.44 %). A significant portion of sequences was identified by IDTAXA/RDP as Acidobacteria (7.4 %/-), but not assigned to this phylum by MR DNA pipeline, being the major discrepancy between the two classification approaches. The other identified phyla contributed to overall bacterial abundance with less than 2.0 %.

Next, we analyzed the most abundant taxa determined in our analysis (i.e. those with relative abundancy higher than 0.5 % of total reads), which accounted for 85.21 %/75.3 % of total reads. As can be seen from Figs. 2 and 59.37 %/43.7 % of total ASVs/zOTUs were resolved to genus level (i. e. with 60 % or higher confidence at genus level/with 95 % or higher homology to queried sequence) and 25.84 %/31.7 % were assigned to lower taxonomy level (i. e. with the confidence less than 60 % at genus level/with lower than 95 % homology to the queried sequence). From the data presented (Fig. 2, greens), we can state that the most abundant genera determined in our sample were *Burkholderia* (40.11 %/28.64 %), *Staphylococcus* (2.78 %/1.7 %), *Ligilactobacilus*/*Lactobacillus* (2.21 %/0.55 %), *Terrimonas* (1.86 %/1.06 %), *Streptococcus* (1.82 %/1.21 %), *Acinetobacter* (1.43 %/0.83 %), *Legionella* (1.21 %/0.71 %), *Pseudomonas* (1.13 %/0.63 %), *Flavobacterium* (0.99 %/0.63 %), *Bifidobacterium* (0.91 %/-), *Sulfuricella* (0.88 %/0.78 %), *Aquicella* (0.87 %/1.66 %), *Sphingomonas* (0.8 %/-), *Lactococcus* (-/1.32 %), *Corynebacterium* (-/1.31 %) and *Rhodoferax* (-/0.76 %). Significant portion of ASVs/zOTUs (25.84 %/31.66 %) was assigned to particular taxonomy level with low homology of query sequence to BLAST results and, therefore, represent unknown genera, i.e. genera that we were not able to identify based on our reference database (Fig. 2, reds).

### 3.3 Isolation of bacterial strains and their taxonomic affiliation

In parallel with sampling the As and Sb rich ochreous environment in front of the mine Budúcnosť for mineralogical and NGS analysis, we also collected a sample intended for the isolation of stable bacterial cultures. For this purpose, we separated a part of pooled ochreous material and from this part we cultivated desired strains. Cultivation was performed either on plates with TSA medium supplemented with standard distilled water, or on TSA medium supplemented with sediment free water collected at the same place as the rest of the sample. The latter was to obtain a cultivation medium enriched in minerals common in the original environment to further support bacterial growth. The selected strains were repeatedly streaked to single colonies to obtain pure cultures. By this approach we collected 59 microbial strains that we were able to keep conserved as glycerol stocks at −80 °C for further analysis. However, we repeatedly failed to sequence 11 of the isolated strains with our primers and because the same strains also did not perform well on media supplemented with antimony, they were excluded from the further analyses.

**Fig. 4:**
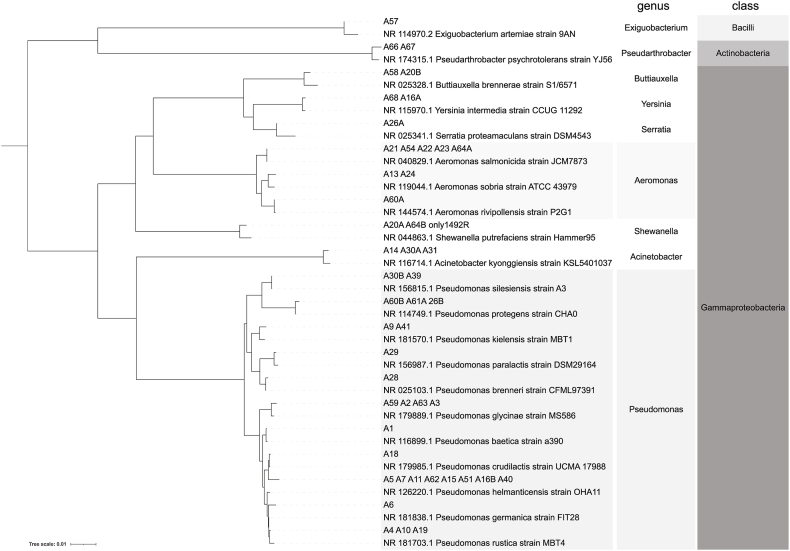
Midpoint rooted Neighbour joining tree with distance correction showing phylogenetic relationships of isolated strains to reference, most related, strains from NCBI 16S ribosomal RNA sequences (Bacteria and Archaea) database and their respective taxonomic affiliation according to IDTAXA/RDP classifier. The leaves with more than one strain represent groups of strains with the identical 16S RNA sequences.

To assign taxonomy to the isolated strains, the 16SRNA V1 – V9 region of each strain was sequenced, manually checked and the identical sequences were grouped together. Representative sequences for each group were BLASTn compared with the NCBI bacterial and archaeal 16S RNA database. In parallel, the sequences were also classified with the IDTAXA/RDP classifier to obtain taxonomy assignments comparable to those generated for NGS data. The full list of isolated strains with their respective 16S RNA sequences and taxonomy classifications is available as a FASTA file in Suppl. D. Then the representative sequences, as in Suppl. D, were aligned and distances calculated by Clustal Omega, and the resulting data were used for Neighbour joining tree construction (Fig. 4). As reference sequences, the most relative and representative bacterial sequences from the NCBI database were used for each group.

The majority of isolated strains were assigned to class Gammaproteobacteria (Proteobacteria) and only three strains were assigned as representatives of other classes, one as the class Bacilli (Firmicutes) and one as the class Acidobacteria (Acidobacteria) (Fig. 4). As can be seen in Fig. 4, the majority of selected strains were identified either as *Pseudomonas* (27) or *Aeromonas* (8), i.e. representatives of two related orders Pseudomonadales and Aeromonadales of phylum Gammaproteobacteria. The sequences identified either as *Pseudomonas* or *Aeromonas* were divergent, and, based on homology comparison, they were sorted to distinct groups. As a result, *Pseudomonas* sequences were grouped into 11 sequence divergent groups (A-K) and *Aeromonas* into 3 groups (A-C). The remaining sequences were assigned as *Acinetobacter* (3, Pseudomonadales), *Buttiauxella* (2, Enterobacterales), *Shewanella* (2, Alteromonadales), *Serratia* (1, Enterobacterales), Yersinia (2, Enterobacterales), *Exiguobacter* (1, Bacillales, Bacilli) and *Pseudarthrobacter* (2, Micrococcales, Acidobacteria) without any further divergences within the groups. In all strains nearly full length 16S RNA gene was sequenced, except *Shewanella* sequences, where sequencing with 27F and 805R primers repeatedly failed, and only the distant part of the gene was used for analysis.

To further support the taxonomic affiliations of isolated strains, basic culture morphology of individual strains was examined by means of color, surface and texture. The observed characteristics are listed in Table 3 and they well support the sequence-based sorting of the isolated strains to individual taxonomic groups.

**Table 3:** List of isolated strains with their taxonomic affiliations based on V1-V9 16S rRNA sequences and their growth characteristics on TSA media supplemented with Sb compared to TSA media and their growth characteristics in liquid TS media supplemented with Sb compared to TSA media. Included are also their morphological characteristics. C means that the strain grows under the tested conditions comparably to the control conditions without Sb, S means that the strain grows slower than at the control conditions, S2 means that the strain grows well, but slower than at the control conditions, S1 means that the strain grows very slowly compared to the control conditions and NO means that the strain does not grow at all or almost at all under the tested conditions.

clone	drop tests on TSA plates			morphology on TSA plates			growth curves in liquid TS			
	100 mg/l Sb	500 mg/l Sb	1000 mg/l Sb	Colour	Surface	Texture	75 mg/l Sb	150 mg/l Sb	300 mg/l Sb	600 mg/l Sb
**Pseudomonas group A**										
**A5**	C	NO	NO	pink/white	glistening	mucoid	C	C	C	S2
**A16B**	NO	NO	NO	pink/white	glistening	mucoid	C	C	C	C
**A7**	C	NO	NO	pink/white	glistening	mucoid				
**A11**	C	NO	NO	pink/white	glistening	mucoid				
**A62**	NO	NO	NO	pink/white	glistening	mucoid				
**A40**	NO	NO	NO	pink/white	glistening	mucoid				
**A15**	C	S	S	pink/white	glistening	mucoid				
**A51**	C	C	C	pink/white	glistening	mucoid				
**Pseudomonas group B**										
**A60B**	C	C	C	slightly yellow	glistening	moist	C	C	C	S1-2
**A61**	C	C	C	slightly yellow	glistening	moist				
**A26B**	C	C	C	slightly yellow	glistening	moist				
**Pseudomonas group C**										
**A9**	C	C	C	slightly yellow	rough	moist	unmeasurable			
**A41**	S	NO	S	slightly yellow	rough	moist				
**Pseudomonas group D**										
**A4**	C	NO	NO	pink/white	glistening	mucoid	C	S1-2	NO-S1	NO
**A10**	C	NO	NO	pink/white	glistening	mucoid				
**A19**	C	C	C	pink/white	glistening	mucoid	C	C	S2	S1-2
**Pseudomonas group E**										
**A30B**	C	C	C	pink/white	glistening	mucoid	C	C	C	S2
**A39**	C	S	S	pink/white	glistening	mucoid				
**Pseudomonas group F**										
**A59**	C	C	C	pink/white	glistening	mucoid	C	C	C	S2
**A2**	C	C	C	pink/white	glistening	mucoid				
**A63**	C	C	C	pink/white	glistening	mucoid				
**A3**	C	C	C	pink/white	glistening	mucoid				
**Pseudomonas group G**										
**A1**	C	NO	NO	pink/white	glistening	mucoid	C	S1-2	NO	NO
**Pseudomonas group H**										
**A6**	C	S	C	pink/white	glistening	mucoid	C	C	C	S2
**Pseudomonas group I**										
**A29**	C	S	C	pink	smooth	moist	C	C	S2	S1
**Pseudomonas group J**										
**A18**	C	NO	NO	pink/white	glistening	mucoid				
**Pseudomonas group K**										
**A28**	C	C	C	slightly yellow	rough	moist	C	C	C	S1-2
**Aeromonas group A**										
**A21**	C	C	C	slightly yellow	glistening	moist	C	C	C	S2
**A54**	C	C	C	slightly yellow	glistening	moist				
**A64A**	C	C	C	slightly yellow	glistening	moist	C	C	C	S1
**A22**	C	C	C	slightly yellow	glistening	moist				
**A23**	C	C	C	slightly yellow	glistening	moist				
**Aeromonas group B**										
**A24**	C	C	C	slightly yellow	glistening	moist	C	C	C	S1
**A13**	C	C	C	slightly yellow	glistening	moist	C	C	C	S2
**Aeromonas group C**										
**A60A**	C	C	C	pink/white	glistening	moist	C	C	C	S1-NO
**Acinetobacter**										
**A14**	C	C	C	brownish	glistening	moist	C	C	C	C
**A30A**	C	C	C	brownish	glistening	moist	C	C	C	C
**A31**	C	C	C	brownish	glistening	moist				
**Buttiauxella**										
**A58**	C	C	C	slightly yellow	glistening	moist	C	C	C	S2
**A20B**	C	C	C	slightly yellow	glistening	moist	C	C	C	S1-NO
**Shewanella**										
**A20A**	C	C	C	pink	glistening	moist	C	C	C	S1
**A64B**	C	C	C	pink	glistening	moist	C	C	C	S2
**Serratia**										
**A26A**	C	C	C	white	smooth	moist	C	C	C	S2
**Exiguobacter**										
**A57**	C	C	C	orange	smooth	moist	C	C	S2	S1
**Yersinia**										
**A68**	C	C	C	slightly yellow	glistening	moist	C	C	C	S1-2
**A16A**	C	C	C	slightly yellow	glistening	moist	C	C	C	S1-2
**Pseudarthrobacter**										
**A66**	C	NO	NO	white	smooth	moist	C	S2	S2	S1-2
**A67**	C	NO	NO	white	smooth	moist				

### 3.4 Estimation of As and Sb tolerance

**Fig. 5:**
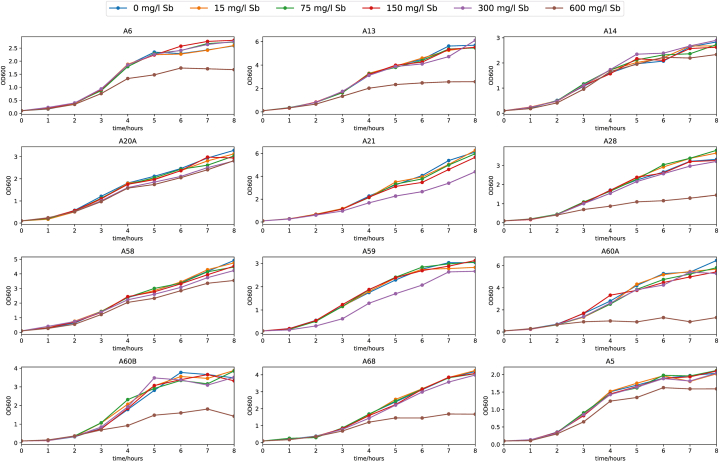
Representative growth curves of the selected, best growing strains, considered to be Sb resistant. The strains were grown in liquid TS media, either not supplemented (control), or supplemented with increasing concentrations of Sb. The concentrations of Sb were measured post experiment and averaged, with the SD error rate 37.1 mg/l Sb at 300 mg/l of Sb.

Next, we were interested in growth performances of selected strains in the presence of elevated concentrations of Sb to select for tolerant or resistant strains. For this purpose, we performed two assays: drop test on TSA plates supplemented with 0, 0.1, 0.5 and 1 g/l of Sb and OD_600_ timepoint measurements in liquid TSA media supplemented with either 0, 15, 75, 150, 300, or 600 mg/l of Sb (these are average concentrations calculated based on ICP-MS measurements of 300 mg/l solution). The results of these two assays are compiled in Table 3 and growth curves of selected strains are available in Fig. 5. The strains growing under elevated Sb concentrations in a manner comparable to the conditions without Sb (control condition) are marked as C (growing as control), those growing slower than control as S (slow growing, S2 - almost like control, S1 - growing very slowly), and those not growing or almost not growing as NO (not growing). First, we performed drop test, and because we were mostly interested in strains resistant to high concentrations of Sb, we selected strains with the best growth capabilities in this assay for the experiments in liquid media (Table 3), but into the growth assay experiments we also included some of the strains not resistant to Sb as negative controls. To reduce the number of strains tested, we always selected only one representative strain for each taxonomical and morphological group where appropriate. Based on the results of growth assays, best performing strains in liquid TS media supplemented with 300 mg/l of Sb were selected and they create an Sb resistant subcollection of isolated strains (Table 4). “Sb resistant” here means that they can grow in TS medium supplemented with 300 mg/l of Sb (as KSb(OH)_6_) to the same, or comparable, level as in Sb free TS medium, however, certain slowdown in growth performance may be observed at Sb concentration above this value. All of these strains were growing on TSA media supplemented with 1 g/l of Sb in the form of KSb(OH)_6_ comparably to the Sb free TSA media. The strains from this subcollection include strains *Pseudomonas* A60B, *Pseudomonas* A59, *Pseudomonas* A28, *Aeromonas* A21, *Aeromonas* A13, *Aeromonas* A60A, *Acinetobacter* A14, *Buttiauxella* A58, *Shewanella* A20A a *Yersinia* A68.

**Table 4:** Average concentrations of Sb in dried cell mass from the Sb removal experiments, where TS represents negative control without the addition of Sb to the cultivation media, TS + Sb represents the experiment where the Sb was added to TS to the final concentration of 300 mg/l ± 37.1 mg/l SD, fold increase represents the calculated fold increase in Sb concentration in TS + Sb media over the control TS media and CFU represents percentage of surviving cells in TS + Sb media compared to control TS media at the end of experiment, pellet means the obtained dried cell mass in each individual experiment.

taxa	clone	TS [mg/kg]	TS + Sb [mg/kg]	fold increase	CFU	TS [ng/pelet]	TS + Sb [ng/pelet]	fold increase
Pseudomonas group A	**A5**	10.42	210.39	20.19	**0.16**	1.85	30.66	16.56
Pseudomonas group B	**A60B**	8.80	**551.80**	62.73	0.94	1.84	**100.12**	54.41
Pseudomonas group F	**A59**	10.55	306.26	29.04	0.90	1.68	46.30	27.57
Pseudomonas group H	**A6**	7.65	377.82	49.37	**0.20**	2.70	40.36	14.97
Pseudomonas group K	**A28**	5.60	247.20	44.14	1.04	1.48	58.69	39.70
Aeromonas group A	**A21**	4.90	**489.93**	100.01	1.24	1.11	**97.46**	87.64
Aeromonas group B	**A13**	14.62	287.77	19.69	0.83	4.25	66.89	15.73
Aeromonas group C	**A60A**	5.31	**639.19**	120.36	0.83	1.47	**118.79**	80.91
Acinetobacter	**A14**	4.88	251.38	51.50	1.10	1.77	24.96	14.08
Buttiauxella	**A58**	2.75	**583.64**	212.07	0.97	0.68	**133.49**	195.50
Shewanella	**A20A**	3.45	**961.28**	278.63	1.07	0.71	**189.04**	267.85
Yersinia	**A68**	3.26	**577.42**	176.94	0.82	0.86	**141.87**	164.94

The Sb was supplemented as KSb(OH)_6_ in both assays to mimic the Sb^V^ oxidation state, i.e. oxidation state common in the environment of iron ochres from which the strains were isolated. However, due to its peculiar nature in terms of solubility, the results of growth assays must be interpreted carefully. The problem here, that we encountered, is that KSb(OH)_6_ is poorly soluble in our water based media and water as well (see). Although we were able to solubilize it by sonication in liquid TSA medium, the crystals reappeared in times interfering with our experimental design at room temperature. We also experimented with decreasing the pH of the solutions and with testing the KOH based water solution as the solubilizer, but without the significant effect upon KSb(OH)_6_ solubility. Finally, we decided to prepare the liquid media supplemented with KSb(OH)_6_ by solubilizing KSb(OH)_6_ in TS to prepare (4 g/l Sb) stock solution. This stock solution was equalized prior to further usage for one day at room temperature. Then the undissolved crystals were removed by filter sterilization and the resulting stock solution was added to TSA media at desired concentrations. Based on ICP-MS measurements we estimated that approximately 15 wt % of KSb(OH)_6_ remains solubilized in TS by this approach, however, the exact amount of Sb in each individual experiment is not known prior the experiment and can be only approximated or measured at the end of the experiment. However, using this approach, we met the purpose of this assay, i.e. to distinguish different growth abilities of individual strains. Nevertheless, powdered KSb(OH)_6_ was added to the agar media directly, and, therefore, the Sb concentrations were as intended. Although some level of KSb(OH)_6_ recrystallization appeared also on the plates, the bacterial growth performances were clearly changing with the increasing Sb concentrations, as expected. And finally, although the addition of KSb(OH)_6_ is expected to increase the pH of media (in our hands ranging from pH 7.0 (0 g/l Sb) to pH 7.9 (4 g/l Sb) in TSA media), we decided not to buffer our media, as the pH differences in our experiments would be only very subtle.

### 3.5 Extraction of Sb from media by isolates

To measure the ability of selected Sb resistant strains to extract Sb from cultivation media, we cultivated the strains in TS media supplemented with 300 mg/l of Sb in the form of KSb(OH)_6_ for 16 h, and then collected the bacterial cells. The concentration of Sb in washed and dried cells was measured by ICP-MS, compared to controls, i.e. the same strains cultivated in TS medium without Sb for 16 h, and expressed as experiment/control ratio (fold increase in Table 4). In parallel, the CFU ability of the strains was determined and compared to controls to reveal viability of cells treated with increased concentration of antimony after 16 h of incubation. The viability of tested strains seems not to be significantly affected by Sb, except that it is significantly reduced in strains *Pseudomonas* A5 and *Pseudomonas* A6. Based on the results of ICP-MS measurements from Table 4, we must state that all assayed strains can retain Sb from cultivation media to certain level, ranging from 210.39 to 961.28 Sb [mg]/dried biomass [kg], but strains *Shewanella* A20A, *Buttiauxella* A58, *Yersinia* A68 and *Aeromonas* A60A can retain SB from media with higher efficiency (ranging from 577.42 -to 961.28 Sb [mg]/dried biomass [kg]) than the other strains involved. Because it is not easy to set appropriate Sb resistant and non-retaining control for this experiment, the strains *Pseudomonas* A5 and *Pseudomonas* A6, growing, but barely surviving under the condition of elevated Sb concentration and retaining Sb to the least extend, should be considered as an alternative to negative control in this experiment. The ability of aforementioned strains to retain Sb more efficiently is even more obvious if the Sb accumulation is recalculated to real amount of dried biomass obtained in each individual experiment (pellet), as this approach includes the differences in growth capabilities of individual strains (see Table 4, last 3 columns). This is because some strains, including *Pseudomonas* A5 and *Pseudomonas* A6 grow slower and produce less biomass in the experimental period of 16 h as the others.

## 4 Discussion

The permitted maximum concentration of Sb in drinking water has been set by the World Health Organization at 5 μg/l [79] and by Slovak legislative at 25 μg/kg. The measured average concentrations 130 μg/l Sb in water at the examined site exceed the Slovak legislative limit more than 5 times. Even more alarming is the situation in solid iron ochre phase where the concentration of Sb reaches 2317 mg/kg Sb in average and is almost 100 times higher than Slovak legislative limit. This reflects the ability of iron ochres to scavenge the polluting metal(loid)s from the environment, an ability that is often implemented in various semi natural bioremediation applications. However, in uncontrolled iron-ochres reservoirs it represents tremendous environmental burden, as iron ochres can be easily drifted to water acceptors by heavy rains. Despite the high concentrations of polluting metal(loid)s, the iron ochres are bursting with microbial life [10], which have an indispensable role in their genesis. However, the role of microbes and the detailed molecular mechanism in the dissolution and oxidation of antimony in Sb-containing minerals and pristine rocks remain poorly understood [18].

V4 16S RNA sequencing is a standard approach to identify bacteria inhabiting environmental samples at the genus level or higher. It has a significant bottleneck in the taxonomy assignment step that relies at the accuracy of the reference 16S RNA database used at a time and the at the algorithm applied to find best matches. Here we compared taxonomy assignments performed with several publicly available 16S RNA databases and different classifiers with the commercially available taxonomy assignment provided by MR DNA. There are substantial differences in how the 16S RNA databases are created and curated and together with systematic errors that exist in various reference databases [[80], [81], [82], [83]] very likely result in different outcomes of individual approaches. Considering the specific environment of iron ochres also proportional contribution of individual environment specific taxa in individual databases must be considered as it might differ significantly.

Comparable total portions of ASVs, not represented by more than 0.5 % abundances in individual samples, were obtained using different bioinformatic approaches (Fig. 2, Suppl. B.), ranging from 13.4 % to 19.4 %, and were not considered further. IDTAXA search algorithm in combination with curated databases GTDB and SILVA resulted in the smallest portions of assigned sequences, 10.32 % and 21.10 % respectively, compared with the other databases, and significant portions of the sequences, 43.2 % and 28.39 % respectively, were not assigned to genus taxonomy level, but only to higher taxonomy levels. GTDB is based on Genebank and RefSeq and follows the List of Prokaryotic names with Standing in Nomenclature (LPSN) [84] nomenclature (https://gtdb.ecogenomic.org/about). The SILVA database builds on phylogeny of 16S rRNA and the taxonomy is based on Bergey's Manual of Systematic Bacteriology [85], GTDB, LPSN, USERS, NCBI for cultured strains and on USERS, SILVA nomenclature for uncultured strains (https://www.arb-silva.de/documentation/silva-taxonomy/), keeping the order in priority.

The best results were obtained with IDTAXA/CONTAX approach (60.91 %/5.38 %) and IDTAXA/RDP approach (59.37 %/2.52 %), where the numbers represent percentage of ASVs taxonomy-assigned to genus level/percentage of unclassified abundant ASVs (Bacteria). The CONTAX database lists millions of classified 16S rRNA sequences obtained from various sources and it is created based on some degree of consensus on the classification [72,82]. The RDP database [86] contains 16S rRNA sequences from the International Nucleotide Sequence Database Collaboration (INSDC) [87] databases and the taxonomy is based on Bergey's Manual of Systematic Bacteriology and LPSN. However, the performances of individual databases on environmental samples cannot be generalized, as they commonly result in different outcome performances, based on sequence dataset and classifier used. For example, the output of RDP classifier (Suppl. B.), using the same RDP database was, clearly different from IDTAXA/RDP approach, as it resulted only in 38.26 % ASVs identified at genus level and in 20.41 % ASVs of unidentified ASVs. Opposing our results, Ramakodi [82] reported SILVA database as the best performing option in combination with IDTAXA classifier for randomly selected soil samples compared to RDP, GTDB and CONTAX. Here we selected IDTAXA/RDP as the best classification option for assigning taxonomies to bacteria isolated from iron ochres, combining good assigning performance (59.37 % assigned ASVs at genus level and only 2.52 % of unassigned ASVs) and highly trusted Bergey's taxonomy. We compared it to commercially obtained MR DNA taxonomy profile (43.66 % at genus level/only 0.97 % as Bacteria), and as can be seen from Fig. 3, Fig. 2, obtained taxonomy profiles are highly similar and can be analyzed in concordance.

Regardless of approach used (Fig. 2, Suppl. B. and Table 2), ASVs referenced as *Burkholderia* (Burkholderiace, Gammaproteobacteria) were the most abundant at this site. The genus *Burkholderia* is widespread in diverse ecological environments, and the majority of known species are soil bacteria interacting with plants. *Burkholderia* is commonly reported from arsenic and antimony polluted environments, including iron ochres, and several arsenic tolerant strains were isolated [[88], [89], [90], [91], [92]], however, no antimony resistant isolate has been reported yet. Analysis of metagenomic-assembled genomes revealed that Burkholderiales likely possess enzyme complexes predicted to be utilized for antimonate reduction [93] and Yang et al. [94] predicted *Burkholderia* to be involved in the reduction or methylation of As in the rhizosphere of paddy soils, possibly via acetic acid and oxalic acid mediated reduction and secondary mineral formation. Li et al [27] predicted *Burkohderia* to be one of the species highly tolerant to high doses of arsenic and antimony co-contamination. There are numerous reports on various *Burkholderia* strains producing metal(loid)s and phosphate bioleaching siderophores and organic acids [88,90,[91], [95]]. Bhakat et al. [90] and Rombola et al. [95] independently reported that the metal(loid) solubilizing activity of *Burkholderia* might be inhibited by As^V^. Mailloux et al. [96] suggested that *Burkholderia fungorum* mobilizes ancillary arsenic from apatite and is partially responsible for As water contamination in Himalayan basin. All together genus *Burkholderia* includes heterotrophic and chemolitotrophic strains with well documented role in bioleaching and mobilization of various metal(loid)s, including As, however, its possible involvement in Sb geocycling remains elusive. Yet, some predictive models suggest that at least some strains might be involved in Sb and As reduction, but experimental evidence for this proposal is missing.

Also, some other abundant candidate Sb tolerant bacterial genera identified at genus level are known to be involved in metal(loid) geocycling. For example, *Rhodoferax ferrireducens* was characterized as a facultatively anaerobic, non-phototrophic, metabolically versatile, Fe^III^ reducing (respiring) bacterium, that can also use nitrate as an electron acceptor [97] and *Rhodoferax* sp. was identified as one of the principal components of arsenic and antimony co-contaminated iron ochres [10]. *Pseudomonas aeruginosa* CA207Ni, *Burkholderia cepacia* CA96Co, *Rhodococcus* sp. AL03Ni, and *Corynebacterium kutscheri* FL108Hg were studied for their ability to effectively accumulate and remove Ni, Co, Cr and Cd metals from aqueous medium [98].

*Lactobacillus* sp.-mediated biosynthesis of Sb_2_O_3_ nanoparticles from SbCl_3_ was reported by Jha et al. [99]. *Terrimonas* sp. was reported to be enriched in hydrogen autotrophic Sb^V^ reducing reaction system in the presence of increased concentrations of nitrate, where its abundance negatively correlated with Sb^V^ reduction [100]. Arsenate, arsenite, and antimonite resistance region on plasmids of *Staphylococcus xylosus* and *Corynebacterium flaccumfaciens* subsp. oortii CO101 was studied extensively [101,102] and *Lactobacillus*, *Flavobacterium* and *Acinetobacter* were reported as the dominant species from watershed of an active Sb mine [25]. Li et al. [20] isolated *Flavobacterium potami* strain resistant to multiple metals, including arsenic and antimony and Jenkins et al. [103] reported *Flavobacterium* sp. to methylate As^III^.

Cultivable aerobic bacteria represent only a fraction of the total microbial diversity in iron ochres and in the environmental samples in general, however, they are still highly important in studies of metal(loid) tolerance, speciation and mobility. Only two abundant bacterial genera identified by the NGS approach, *Pseudomonas* and *Acinetobacter*, were also cultivable in the laboratory in our conditions. Out of 48 isolated strains, we were able to select 10 Sb resistant strains, while each of them is a representative of an unique bacterial genus or of a group within the genus. All these strains were able to retain Sb from cultivation media to a certain extent by an undetermined mechanism, possibly by employing EPSs, metallothionein substances or bioprecipitation.

Metallothioneins are low molecular weight, Cys-rich, cadmium, zinc and copper binding proteins [104]. The reports of bacterial metallothioneins in metal binding are only sparse and up to the date only three bacterial metallothioneins were investigated. Metallothionein SmtA from the Gram-negative cyanobacterium *Synechococcus elongatus* is important in zinc detoxification [105,106], while metallothionein MymT from the pathogenic Gram-positive bacterium *Mycobacterium tuberculosis* is crucial for copper detoxification [107]. Recently, it has been reported that *Pseudomonas fluorescence* Q2-87 produces metallothionein PflQ2 important for zinc homeostasis and cadmium detoxification, but it is expressed mainly in the stationary phase, and, therefore, possibly has a specific role in starvation survival [51]. Metallothioneins are conserved in 90 % of newly sequenced bacterial *Pseudomonas* genomes, and it is therefore tempting to speculate, that the Sb resistant *Pseudomonas* strains isolated from iron ochres might adopt similar strategy to cope with high doses of metal(loid)s, including antimony. However, more experiments to address this possibility are needed.

Reports on bacterial EPSs are more common ([52] and references therein) and they were also reported as Cu, Cd, Zn, Ni binding substances in *Pseudomonas* CU-1 [108] and Zn and Pb binding substances in *Shewanella oneidensis* MR-1 [109], however, no EPS binding of antimony was reported up to date.

Another mode of bacterial interaction with antimony resulting in antimony retention from media or environmnet is antimony precipitation in the form of insoluble biominerals. Liu et al. [37] reported that *Shewanella putrefaciens* IAR-S1 and *S. xiamenensis* IR-S2 re-sequestrated ferrihydrite bound As to new minerals vivianite and magnetite with the assistance of EPSs and Jia et al. [59] reported that Sb^V^-O-Fe^III^ secondary mineral was produced by *Shewanella* in the presence of Fe^III^. *Shewanella* sp. CNZ-1 was shown to reduce Sb^V^ to Sb^III^ and in the presence of SO_3_^2−^ and S_2_O_3_^2−^ to precipitate Sb_2_S_3_ and Sb_2_O_3_ minerals [55]. Similarly, *Pseudomonas* was enriched in antimony acclimated and sulfate rich wastewater cultures precipitating Sb_2_O_3_ or Sb_2_S [58].

Methalothioneins, EPSs, but also bioprecipitation can alleviate metal(loid) concentration burden, especially at high concentrations, and represent an effective survival strategy. Other mechanisms required for Sb resistance are intracellular Sb^V^ reduction, trivalent metalloid/H^+^ antiporter activity pumping Sb^III^ out of the cells and Sb methylation and they are effective especially at lower metal(loid) concentrations.

In respect to Sb mobilization in iron ochres via changing its redox state, from herein isolated bacteria, only certain *Pseudomonas* [[110], [111], [112]] and *Acinetobacter* [8,42] strains were reported to be able to oxidize Sb, despite the fact that reports of Sb oxidizing bacteria are relatively abundant. Sb resistance encoded by *ars* operon was reported for *Pseudomonas aeruginosa* [113] and for Sb^III^-resistant bacterium *Acinetobacter johnsonii* JH7 [53,[112], [114]]. Several *Acinetobacter* strains are also well known for their multiple metal(loid)s tolerance and/or arsenite oxidation [[115], [116], [117], [118]]. The reports of antimony reducing bacteria are generally sparse, but, among them *Shewanella* has been recognized as a model dissimilatory metal reducing bacterium for studying extracellular electron transfer to various metals, metalloids and their minerals, including electroconductive antimony-doped tin oxide nanoparticles [119,120] and it is very likely that it can use antimonate as an electron acceptor as well.

The other bacterial genera identified among the isolated strains have no assigned role in antimony geocycling and/or accumulation up to date. However, the *Buttiauxella* sp. SaSR13 associated with rhizosphere was reported to enhance cadmium accumulation of an hyperaccumulator *Sedum alfredii* plants [121]. Haris et al. [122] used non/living biomass of arsenite-resistant psychrotolerant bacterial strain *Yersinia* sp. strain SOM-12D3 for arsenic biosorption and also strains of *Yersinia enterocolitica* and *Yersinia intermedia* resistant to multiple metal(loid)s (including arsenic) were isolated [123]. Ability of *Aeromonas* to reduce As^V^ in dissimilatory pathway is well studied [124,[125], [126]], and in some strains it is also coupled with multiple metal(loid)s resistance. For the *Aeromonas* strain O23A even production of siderophores and biofilm production were reported [126]. Lukasz et al. [127] isolated As resistant strains *Shewanella* sp. OM1, *Pseudomonas* sp. OM2, *Aeromonas* sp. OM4, and *Serratia* sp. OM17 from an old gold mine in Poland. All of those strains were able to produce mineral solubilizing siderophores and to reduce As^V^ in dissimilatory pathways.

Two bacterial strains, *Pseudarthrobacter* and *Exiguobacterium*, isolated from iron ochres herein were not included in Sb accumulation assay, as their ability to grow in liquid TS media supplemented with 600 mg/l, 300 mg/l and even 150 mg/l was significantly reduced, however both strains performed well in our drop tests on TSA + Sb plates. Although there aren't any reports of *Exiguobacterium* interacting with antimony, several As resistant strains have been reported up to date. Furthermore, *Exiuobacterium* sp. WK6 was reported to reduce arsenate via a non-respiratory metabolic pathway [124] and *Exiguobacterium profundum* PT2 reported to reduce arsenate and remove it from wastewater by biosorption [128]. But also, arsenite oxidizing strain *Exiguobacterium* sp. As-9, efficiently removing arsenite from water, has been reported [129,130]. *Pseudarthrobacter* was reported as Sb oxidizing bacterium by Li et al. [42], however without any references to experimental evidence. One strain *Pseudarthrobacter* sp. AG30 was isolated from old gold and copper mine and its draft genome sequence has been published and numerous genes were reported that could be putatively involved in heavy metal tolerance, including ars genes [131] and some other *Pseudarthrobacter* strains were reported from heavy metal(loid)s polluted areas [132,133].

## 5 Conclusions

The results of bacterial strains isolation and bacterial microbiome characterization from naturally occurring hydrous ferric oxides at Sb contaminated site nearby abandoned mine Budúcnosť (Pezinok, western Slovakia) were summarized and compared. Based on V4 16S RNA NGS approach, numerous bacterial sequences were identified to varying levels of taxonomic ranking. To increase reliability of V4 16S RNA taxonomy assignments the obtained sequences were clustered, filtered and denoised by two independent approaches and different taxonomy classifiers were applied and compared. The tested methods resulted in certain differences in assigned taxonomies and highlight the importance of appropriate bioinformatic approach selection. As the best performing approach Qiime2 pipeline combined with IDTAXA/RDP taxonomy was selected and presented together with the results of commercial MR DNA taxonomy analysis pipeline. Many of bacteria identified by V4 16S RNA NGS approach are well-known extremophilic bacteria involved in geocycling of metal(loid)s, including antimony. Only a part of the population identified by V4 16S RNA NGS approach was cultivable under the standard laboratory conditions. Among the isolated bacteria there were also strains belonging to genera already known to be metabolically active over the antimony speciation, and hence, also the antimony geocycling (e.g. *Acinetobacter*, *Pseudomonas*, *Shewanella*), but there were also strains with no reported activity towards Sb (*Aeromonas*, *Buttiauxella*, *Pseudarthrobacter*, *Yersinia* and *Exiguobacterium*). In total 48 bacterial strains obtained from the same iron ochre site were tested against their ability to survive in cultures containing high levels of antimony. The best performing strains, growing on media highly exceeding the standard limit values of Sb in the environment, were selected and their ability to retain antimony from the medium has been evaluated. Based on the results of ICP-MS measurements, seven bacterial strains (*Aeromonas* A21, *Aeromonas* A13, *Aeromonas* A60A, *Acinetobacter* A14, *Buttiauxella* A58, *Shewanella* A20A and *Yersinia* A68) were selected that can retain Sb from media with higher efficiency than the other strains involved. Although further experiments are needed, it is possible that the isolated bacterial strains may be useful for bioremediation of antimony from a contaminated environment or for laboratory testing of Sb mineral biotransformation and Sb speciation.

## CRediT authorship contribution statement

**Hana Majerová:** Conceptualization, Formal analysis, Investigation, Methodology, Software, Visualization, Writing – original draft. **Zuzana Konyariková:** Data curation, Investigation. **Dana Strašiftáková:** Data curation, Formal analysis. **Christian Puhr:** Formal analysis, Investigation. **Ivona Kautmanová:** Conceptualization, Funding acquisition, Project administration, Resources. **Tomáš Faragó:** Formal analysis, Investigation, Validation, Visualization, Writing – review & editing. **Peter Šottník:** Conceptualization, Funding acquisition, Project administration, Resources. **Bronislava Lalinská-Voleková:** Conceptualization, Funding acquisition, Methodology, Project administration, Resources, Supervision, Writing – review & editing.

## Funding sources

This work was supported by the Grant Agency for Research and Development with project numbers: APVV-21-0212; APVV-17-0317; the Operational Program of Integrated Infrastructure: “DNA barcoding of Slovakia (SK-BOL), as a part of international initiative International Barcode of Life (iBOL)” (ITMS2014 + 313021W683) and the Environmental Quality Operational Program: “Ensuring the monitoring of environmental burdens in Slovakia - Part 2”, call code OPKZP-PO1-SC142-2015-4 (ITMS number: 310011AXF2).

## Declaration of competing interest

The authors declare that they have no known competing financial interests or personal relationships that could have appeared to influence the work reported in this paper.

## Figures and Tables

**figs1:**
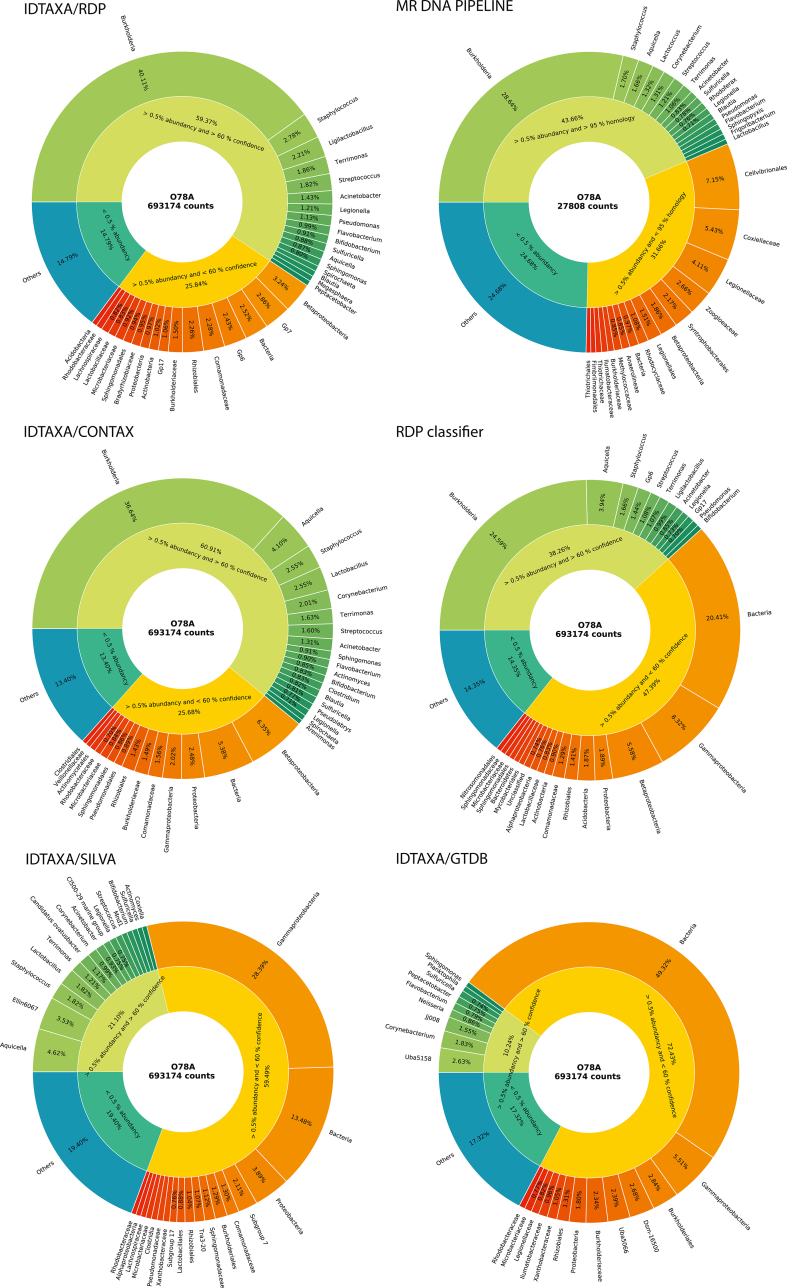
Plots comparing taxonomy generated by different classification approaches. See text for details. Greens show the most abundant genera detected (more than 60% confidence at genera level (IDTAXA/RDP, IDTAXA/GTDB, IDTAXA/SILVA, IDTAXA/CONTAX), more than 80% confidence at genera level (RDP classifier), or more than 95% sequence homology (MR DNA pipeline)) with relative abundancy above 0.5%, reds show the most abundant taxa other than genera with relative abundancy above 0.5%. Blues show all taxonomy units with relative abundancy below 0.5% summed together.
